# If It Looks Like a Duck, Swims Like a Duck, and Quacks Like a Duck—Does It Have to Be a Duck?

**DOI:** 10.1371/journal.pntd.0004430

**Published:** 2016-04-28

**Authors:** Damalie Nalwanga, Lars Henning

**Affiliations:** 1 Infectious Diseases Institute, College of Health Sciences, Makerere University, Kampala, Uganda; 2 Division of Infectious Diseases and Hospital Epidemiology, University Hospital Zurich, University of Zurich, Zurich, Switzerland; University of California San Diego School of Medicine, UNITED STATES

## Case Presentation

**Fig 1:**
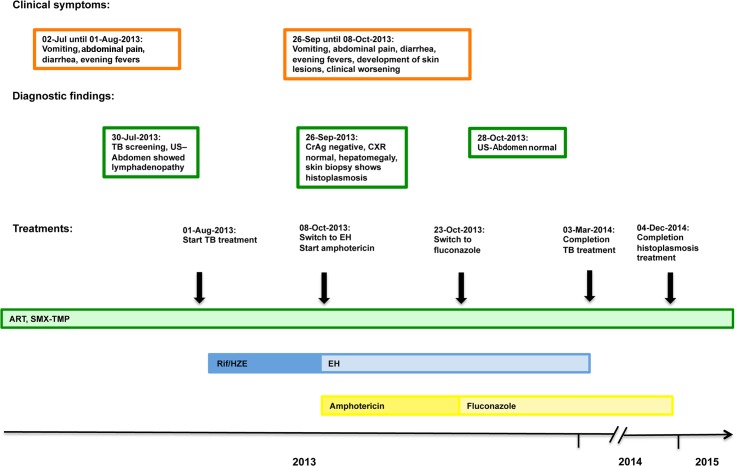
Schematic representation of the patient’s 18-month follow-up with the clinical symptoms, most important diagnostic findings, and treatment timeline. Please note that the scheme is not to scale.

On 2nd July 2013, a 29-year-old HIV-positive woman presented herself to the outpatient clinic at the Infectious Diseases Institute in Kampala, Uganda. Her weight had decreased from 46 kg to 42 kg in the past few weeks. In addition, she complained about abdominal pain, diarrhea, vomiting, and evening fevers during the week leading up to her visit (see Table 1 and Fig 1 for patient characteristics). Her CD4 T cell count in June 2013 was 34 cells/μl, and she had documented second-line antiretroviral treatment (tenofovir disoproxil fumarate, emtricitabine, and lopinavir-ritonavir) failure. She admitted to taking her medications irregularly and was on trimethoprim-sulfamethoxazole prophylaxis. Her last HIV-1 RNA viral load in June 2013 was 199,994 copies/ml. Based on her immunosuppression and symptoms, we screened her for tuberculosis (TB). At the time of screening, she could not produce sputum, but an abdominal ultrasound in late June 2013 showed a lymphadenopathy. A chest X-ray was not carried out at baseline, as it would not have changed the clinical decision to treat the presumptive diagnosis of extrapulmonary TB. Her glomerular filtration rate (GFR) was 55 mL/min, the liver enzyme alanine aminotransferase was 33 IU/L (normal range 0–35 IU/L), and her albumin level was slightly decreased (35.5 g/L; normal range 38–47 g/L).

**Table 1:** Patient characteristics.

Date	Weight (kg)	Medication	Symptoms	Laboratory findings	Remarks
04-Jun-2013	46	ART, TMP-SMX			Routine follow-up
02-Jul-2013	42	ART, TMP-SMX	Vomiting, abdominal pain, diarrhea, evening fevers	CD4: 34 c/μLVL: 199,994 c/ml	
30-Jul-2013	42	ART, TMP-SMX	Vomiting, abdominal pain, diarrhea, evening fevers		TB screening, US; abdomen showed lymphadenopathy
01-Aug-2013	n.d.	ART, TMP-SMX, Rif/HZE	Vomiting, abdominal pain, diarrhea, evening fevers	ALT: 33 IU/LAlbumin: 35.5 g/LGFR: 55 ml/min	Start TB treatment
12-Aug-2013	41	ART, TMP-SMX, Rif/HZE	Clinical improvement		
26-Sep-2013	36	ART, TMP-SMX, Rif/HZE	Vomiting, abdominal pain, diarrhea, development of skin lesions	Serum CrAg negative	Hepatomegaly, CXR normal, skin biopsy shows histoplasmosis
08-Oct-2013	n.d.	ART, TMP-SMX, EH, Ampho	Clinical worsening	ALT: 25 IU/LGFR: 57 ml/min	Switch to EH, start histoplasmosis treatment
28-Oct-2013	n.d.	ART, TMP-SMX, EH, Fluconazole	Generalized body weakness	GFR: 46 ml/min	US; abdomen normal
22-Feb-2014	46	ART, TMP-SMX, EH, Fluconazole	Skin lesions healed	GFR: 50 ml/min	Routine follow-up
03-Mar-2014	48	ART, TMP-SMX, Fluconazole			Completion TB treatment
04-Dec-2014	n.d.	ART, TMP-SMX			Fluconazole stopped
16-Jun-2015	49	ART, TMP-SMX		CD4: 19 c/μLVL: 252,000 c/ml	Routine follow-up

kg, kilogram; ART, antiretroviral treatment; TMP-SMX, trimethoprim-sulphamethoxazole; CD4, CD-4 T cell count; c/μL, cells per microliter; VL, HIV-1 RNA plasma viral load; c/ml, copies per milliliter; TB, tuberculosis; US, ultrasound; n.d., not done; Rif/HZE, rifabutin, isoniazid, pyrazinamide, ethambutol; CXR, chest X-ray; ALT, alanine aminotransferase; IU/L, International Unit per liter; g/L, gram per liter; GFR, glomerular filtration rate; ml/min, milliliter per minute; CrAG, cryptococcal antigen; EH, ethambutol, isoniazid; Ampho, amphotericin B deoxcholeate.

## Progress

**Fig 2:**
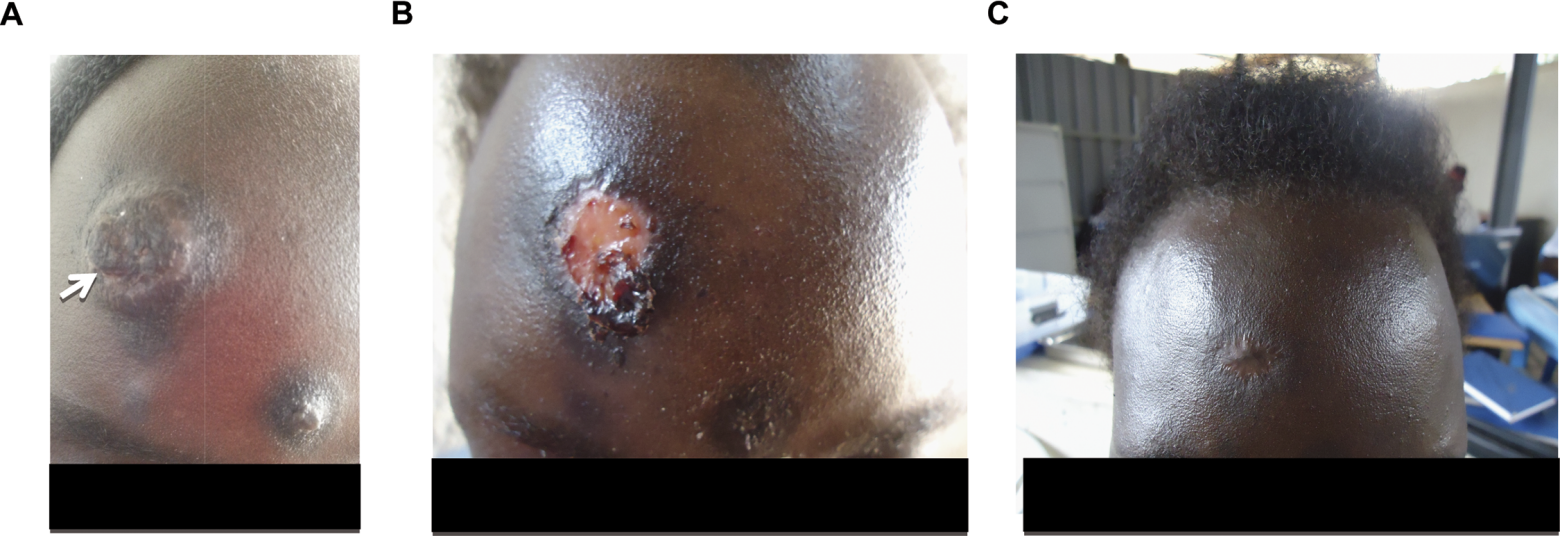
The patient’s skin lesions before and during antifungal treatment. A) Patient presented with skin lesions on 26th September 2013. A biopsy was taken from the large lesion and led to a diagnosis of *Histoplasma capsulatum* (see arrow). B) Clinical response after a 14-day course of amphotericin B deoxycholate (0.7 mg/kg body weight), followed by six days of fluconazole (400 mg twice daily) maintenance therapy. C) Four months into maintenance treatment, the lesions had resolved.

She was started on antituberculosis treatment consisting of rifabutin, isoniazid, ethambutol, and pyrazinamide on 1st August 2013. During follow-up, the patient claimed to adhere to her treatments and reported that the abdominal pain, vomiting, diarrhea, and evening fever stopped; nevertheless, she continued to lose weight in the weeks that followed. At the follow-up visit on 26th September 2013, the patient reported the development of hyperpigmented, progressive nodular, dome-shaped, non-itchy, and well-demarked facial skin lesions that had increased in number and size during the previous four weeks (see Fig 2A). She did not have prior lesions and complained again about abdominal pain, diarrhea, vomiting, and evening fevers. Despite adherence to treatment, she had lost about 15% of her body weight since treatment began. Apart from generalized weakness and a hepatomegaly (approximately 5 centimeters under the right costal margin), no abnormalities were found during physical examination, especially neither palpable lymph nodes nor oral or eye symptoms.

## What Is Your Differential Diagnosis?

Despite the possible explanation of (multi-) drug resistant TB, the continued weight loss and the appearance of new lesions warranted a revision of the initial diagnosis and additional investigations. In this woman, the differential diagnoses included but were not limited to TB of the skin, Kaposi sarcoma, atypical mycobacteria infection, disseminated fungal diseases, and neoplastic mimics of Kaposi sarcoma–like cutaneous T cell lymphoma. The situation was complicated by the fact that the patient had a high HIV-1 viral load at the beginning of TB treatment, and restarting her antiviral treatment together with her TB treatment could have led to clinical deterioration because of immune reconstitution inflammatory syndrome occurrence.

## What Investigations Would You Ask For?

In our resource-limited setting, we opted for a stepwise approach. A chest X-ray was unremarkable (not shown); the serum antigen test for *Cryptococcus neoformans* was negative. A skin biopsy showed *Histoplasmosis capsulatum* infection. We did not have access to *H*. *capsulatum* detection tests. For a review of molecular epidemiology, clinical findings, diagnosis, and treatment of histoplasmosis in HIV-infected patients, please refer to the review of Adenis [1].

## How Would You Manage This Case? Would You Continue TB Treatment?

The woman was hospitalized for treatment of disseminated histoplasmosis. Treatment for severe disseminated histoplasmosis ideally consists of liposomal amphotericin B 3 mg/kg/d for seven to 14 days or until clinical improvement, followed by itraconazole 200 mg three times daily for three days, then 200 mg twice daily for at least 12 months where available [2]. In our setting, only amphotericin B deoxycholate and fluconazole were available. We treated the patient with 0.7 mg/kg amphotericin B deoxycholate for 14 days, then switched to fluconazole 400 mg twice daily for two weeks and afterwards to 400 mg once daily for a maintenance phase.

Because of the initial clinical response to TB treatment, the TB treatment was continued. Based on the Ugandan national guidelines, and to avoid rifampicin-lopinavir/ritonavir interaction, we choose ethambutol/isoniazid for maintenance treatment, although this regime has been shown to be inferior to rifampicin/isoniazid [3].

## Follow-up

The lesions responded very well to amphotericin B deoxycholate treatment, and the patient’s weight began to increase (see Table 1 and Fig 1). A follow-up abdominal ultrasound on 28th October 2013 showed that the lymphadenopathy had been resolved. During the amphotericin B deoxycholate therapy, we regularly monitored serum electrolytes and the GFR. The GFR remained stable over time. Four months into antifungal treatment, the patient reached her baseline body weight of 46 kg and the lesions had healed (see Fig 2B and 2C).

The patient was seen regularly, and the fluconazole was stopped on 4th December 2014. She did not develop any new skin lesions and was last seen on 16th June 2015. On that day, her CD4 T cell count was 19 cells/microliter, and her HIV-1 RNA viral load was 252,000 copies/milliliter. Her body weight was stable at 49 kg, and the patient did not have any clinical complaints. The patient is still on failing second-line antiretroviral treatment and has been enrolled in the control arm of the AIDS Clinical Trial Group Study A5288 (Management Using the Latest Technologies in Resource-Limited Settings to Optimize Combination Therapy After Viral Failure).

## Case Discussion

This case illustrates the challenges of diagnosing (disseminated) histoplasmosis in our particular setting. Without skin involvement, the diagnosis would have been difficult to ascertain, as *H*. *capsulatum* serum antigen tests were not available. The overlap of histoplasmosis and TB clinical findings is shown in Table 2.

**Table 2:** Common clinical symptoms of disseminated histoplasmosis and tuberculosis.

Fever
Weight loss
Lung disease
Hepatosplenomegaly
CNS complaint and/or meningitis
Lymphadenopathy

Data on drug-resistant, extrapulmonary TB in Uganda is only anecdotal, but the first national survey published in 2013 showed that the 1.4% of previously untreated sputum smear–positive TB patients were multidrug-resistant [4]. Because the patient was critically ill and response to TB treatment can take several weeks or even months, we decided to continue TB treatment. In view of the response to the antifungal treatment, continuation of TB treatment should be seen as critical in retrospect.

Because of financial constraints, state-of-the-art maintenance treatment with itraconazole could not be provided. In addition, use of itraconazole warrants pharmacological monitoring, which was not available in our setting. Nevertheless, the patient responded well to fluconazole.

**Key Learning Points**

- Diagnosis of TB can be challenging in resource-limited settings. No “antibiotic trial” for TB diagnosis should be given.
- The clinical symptoms of histoplasmosis and TB overlap, which results in underdiagnosis of histoplasmosis in Africa, but *H*. *capsulatum* polysaccharide can be detected with high sensitivity and specificity in serum and bronchioloalveolar lavages.
- Fluconazole has been shown to be less effective in the maintenance phase than itraconazole in clinical trials but has been used on an ad-hoc basis. A maintenance phase should be carried out for at least 12 months, and lifelong suppressive therapy may be required if immunosuppression cannot be reversed.
- Although consolidation phase treatment with rifampicin/isoniazid has been proven to be superior to ethambutol/isoniazid, this treatment circumvented the hepatotoxic interaction between the protease inhibitor and rifampicin, and eight months of ethambutol/isoniazid continues to be the standard in Uganda.
